# Construction of Hollow TiO_2_/ZnS Heterojunction Photocatalysts for Highly Enhanced Photodegradation of Tetracycline Hydrochloride

**DOI:** 10.3390/molecules30173644

**Published:** 2025-09-07

**Authors:** Ying Zhang, Anhui Su, Yuqin Ding, Yuhan Wu, Yapeng Tan, Jianguo Chang

**Affiliations:** Anhui Provincial Key Laboratory of Green Carbon Chemistry, School of Chemistry and Material Engineering, Fuyang Normal University, Fuyang 236037, China

**Keywords:** hollow TiO_2_, Type-II heterostructure, photocatalytic activity, hydrothermal, tetracycline removal

## Abstract

TiO_2_ photocatalysts exhibit great potential in solar fuel production and environmental remediation, yet their practical applications are often hindered by high electron-hole recombination rates. This study presents a novel strategy for fabricating hollow anatase TiO_2_-modified ZnS heterostructures (TiO_2_/ZnS) via a simple hydrothermal method. The heterostructure effectively combines the high electron mobility of ZnS, which facilitates rapid photogenerated electron transfer, with the high specific surface area of hollow TiO_2,_ which enhances pollutant adsorption. As a result, TiO_2_/ZnS demonstrates superior tetracycline degradation efficiency due to optimized charge separation and improved accessibility to reactive sites, compared to pristine TiO_2_ and ZnS. Furthermore, the enhanced photocatalytic activity is attributed to efficient charge separation facilitated by Type-II heterojunctions between ZnS and anatase TiO_2_. Cycling tests reveal that TiO_2_/ZnS retains over 94% of its activity after 5 cycles. This work offers a versatile approach for stabilizing metal oxides through heterostructure engineering, with significant implications for scalable environmental catalysis.

## 1. Introduction

Tetracycline hydrochloride is one of the most fundamental and widely utilized antibiotics among the tetracycline class of broad-spectrum antibiotics [1]. However, only a fraction of tetracycline is absorbed by humans or animals, while the remaining active pharmaceutical ingredients persist in aquatic and terrestrial ecosystems, leading to substantial ecological harm through the induction of antibiotic resistance and disruption of microbial communities [2]. Traditionally, titanium dioxide (TiO_2_) has been extensively used as a photocatalytic material for tetracycline degradation [3]. Nevertheless, the TiO_2_-based systems that rely on physical adsorption suffer from rapid shifts in adsorption–desorption equilibrium and high regeneration costs, limiting their operational sustainability [4,5]. As a result, advanced photocatalytic technologies have emerged as promising alternatives, offering the dual benefits of solar energy utilization and the complete mineralization of organic pollutants, thus enhancing both green economy metrics and reaction step efficiency [6].

Most reported photocatalytic systems rely predominantly on ultraviolet light irradiation, which constitutes only about 5% of the solar spectrum, leading to significant underutilization of solar energy [7]. In contrast, visible light-driven TiO_2_-based photocatalysts have garnered significant attention as sustainable alternatives, operating under visible light conditions with reduced energy input [8]. However, the intrinsic limitations of TiO_2_—specifically its wide bandgap (anatase phase: 3.2 eV) and suboptimal quantum efficiency (<5%)—severely restrict its photocatalytic activity under visible-light irradiation, resulting in performance that is far from satisfactory [9,10].

To overcome these limitations, considerable efforts have been devoted to developing visible light-responsive TiO_2_-based photocatalysts. Among various strategies, constructing heterojunctions with semiconductors has proven particularly effective [11]. Indeed, highly active co-catalysts, such as metal compounds (e.g., Bi_2_O_3_, ZnO, and ZnS), can form heterojunctions with TiO_2_, thereby generating built-in electric fields that reduce carrier recombination rates and prolong hole lifetimes, thus enhancing the overall photocatalytic efficiency [12,13,14,15,16,17,18]. Therefore, to achieve high efficiency in tetracycline degradation, it is crucial to systematically investigate the electronic band structures of co-catalysts and the interfacial degradation mechanisms between co-catalysts and photocatalysts [19]. However, this remains a significant challenge. Notably, ZnS has emerged as an ideal co-catalyst candidate due to its appropriate band gap (3.7 eV), high conduction band potential, excellent electron mobility, good chemical stability, stable anchoring on support surfaces, and ability to optimize interfacial charge transfer [20,21]. When coupled with ZnS, TiO_2_ can form efficient heterojunctions that not only extend light absorption [22] but also significantly promote the separation of photogenerated charge carriers through built-in electric fields [23]. This synergistic effect addresses the core challenge of rapid electron-hole recombination in pure TiO_2_ while maintaining strong redox capability.

Regretfully, TiO_2_/ZnS heterojunction systems still face critical challenges in photocatalytic antibiotic degradation, including rapid photogenerated hole recombination and unresolved mechanistic details regarding the tetracycline degradation pathway [24]. Moreover, achieving simultaneously enhanced light absorption and charge separation in a rationally designed architecture continues to be a significant materials challenge [25,26].

In this study, we designed and synthesized a novel TiO_2_/ZnS heterojunction photocatalyst with a hollow spherical structure specifically for efficient tetracycline degradation under visible light irradiation. The TiO_2_/ZnS heterojunction catalyst extends the light absorption range of wide-bandgap TiO_2_ while simultaneously suppressing charge carrier recombination in ZnS. Characterization via transmission electron microscopy (TEM) and X-ray photoelectron spectroscopy (XPS) revealed that TiO_2_ exhibited a hollow porous structure, and a distinct heterojunction interface was formed between ZnS and TiO_2_, confirming the successful fabrication of the composite. Performance tests showed that the average carrier lifetimes of photogenerated holes in TZNS were 1.85 and 1.42 times longer than those of pure TiO_2_ and ZnS, respectively, indicating significantly enhanced charge separation efficiency. In situ electron paramagnetic resonance (EPR) analysis revealed that ZnS optimized the driving force of photogenerated holes, promoting hydroxyl radical generation and accelerating tetracycline degradation. Under visible light irradiation (100 min), the optimal sample TZNS-100 achieved 99% tetracycline degradation efficiency, demonstrating excellent photocatalytic activity. This work provides fundamental insight into the design principles of efficient heterojunction photocatalysts for antibiotic wastewater treatment.

## 2. Results and Discussion

### 2.1. Structure Characterization

**Figure 1:**
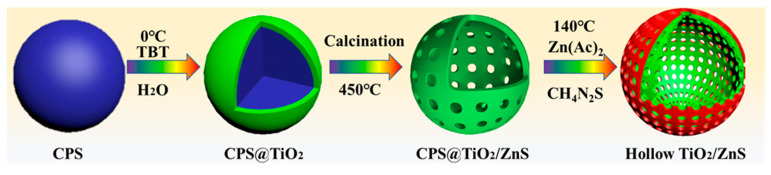
Schematic illustration of the synthesis of TiO_2_/ZnS heterostructures.

The fabrication of the TiO_2_/ZnS heterostructure (denoted TZnS) is systematically illustrated in Figure 1. Synthesis began with the preparation of uniform TiO_2_ nanospheres via controlled polystyrene sphere (CPS) template-directed self-assembly, followed by solvothermal crystallization. Subsequently, the TiO_2_ spheres were purified via centrifugation and vacuum drying, then reacted with zinc acetate dihydrate and thiourea under hydrothermal synthesis at 140 °C for 4 h. This sequential process enabled controlled sulfidation, yielding well-defined TiO_2_/ZnS core–shell heterostructures with hollow TiO_2_ cores and burr-like ZnS shells.

The morphologies of pristine TiO_2_ and ZnS were characterized by TEM (Figures S1 and S2). As shown in Figure S1a, TiO_2_ exhibits well-defined hollow spherical structures (Figures S1a and S2a,b), enhancing pollutant adsorption and providing abundant photocatalytic active sites. HR-TEM analysis (Figure S1e) [27] confirms the (101) lattice planes, consistent with the characteristic 25.6° (101) peak of anatase TiO_2_ (JCPDS: 21-1272) shown in the inset, validating the successful synthesis of hollow TiO_2_ spheres via template-directed self-assembly.

**Figure 2:**
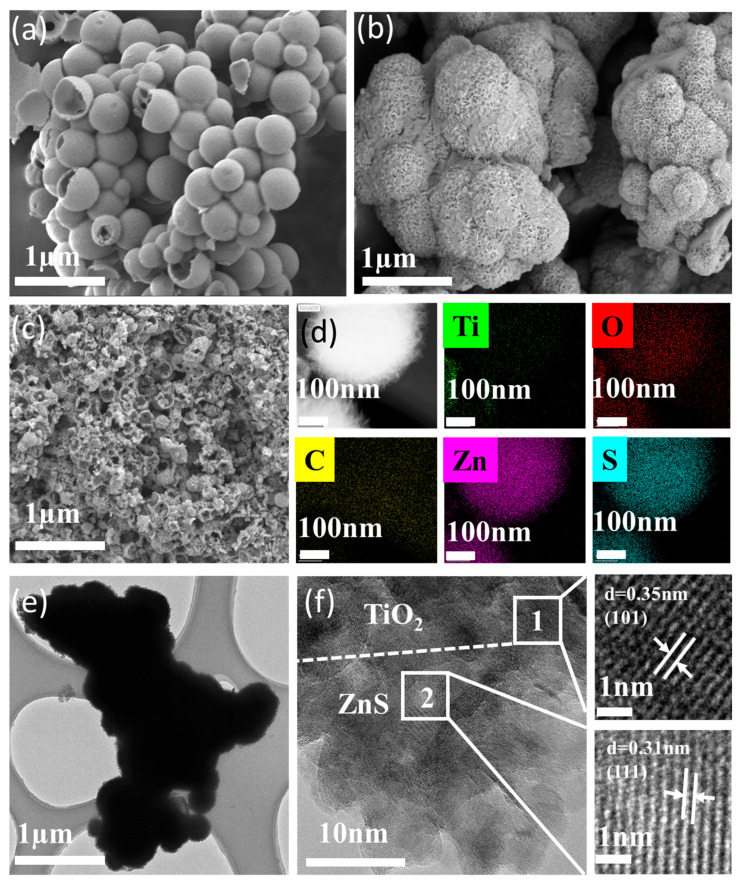
Morphological and Structural Characterization of TiO_2_, ZnS, and TZnS−100. (**a**–**c**) SEM images of (**a**) TiO_2_, (**b**) ZnS and (**c**) TZnS, showing evolution from smooth TiO_2_ spheres to ZnS-decorated TiO_2_ surfaces; (**d**) EDS elemental mapping (Ti, Zn, S, O) of TZnS−100, showing homogeneous ZnS distribution on the TiO_2_ scaffold; (**e**) TEM image of a single TZnS−100 particle; (**f**) HRTEM image of TZnS−100, showing lattice fringes of 0.35 nm (TiO_2_ (101)) and 0.31 nm (ZnS (111)), confirming intimate interfacial contact. All scale bars: (**a**–**c**) 1 μm; (**d**) 100 nm; (**f**) 10 nm, and 1 nm.

TEM characterization of ZnS (Figure S2a) reveals nanoparticulate aggregates with irregular surface morphology, consistent with the SEM image in Figure 2b. The lattice fringe spacing of 0.312 nm in HR-TEM (Figure S2e) corresponds to the (111) planes of cubic ZnS [28]. Furthermore, elemental mapping (Figure S2b–d) reveals uniform Zn and S distribution, confirming successful ZnS synthesis.

SEM and TEM analysis of the TZnS−100 photocatalysts (Figure 2) demonstrates intimate integration of the components. Figure 2d shows ZnS particles densely anchored on the TiO_2_ surface, with elemental mapping confirming uniform Zn and S distribution. HR-TEM (Figure 2f) reveals a lattice spacing of 0.347 nm [29] and 0.312 nm [28], corresponding to the (101) planes of anatase TiO_2_ and (111) planes of ZnS, respectively, confirming ZnS deposition on the TiO_2_ surface and the formation of a TiO_2_/ZnS N-N heterostructure.

**Figure 3:**
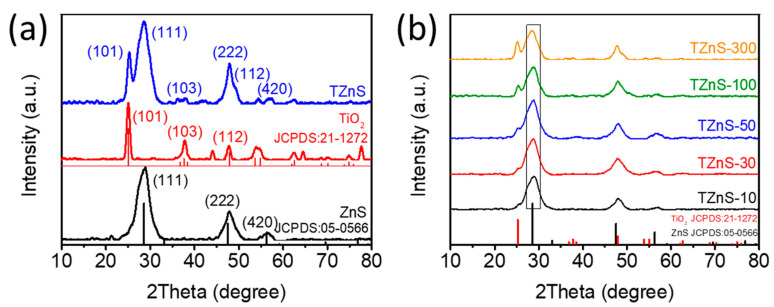
XRD patterns of TiO_2_, ZnS, and TZnS: (**a**) TZnS−100, TiO_2,_ and ZnS; (**b**) TZnS-X with varying TiO_2_, mass percentage (X denotes the mass percentage of TiO_2_).

X-ray diffraction (XRD) analysis was performed on the as-synthesized materials (Figure 3). The XRD pattern of pristine ZnS exhibits diffraction peaks at 28.89°, 47.91°, and 56.62°, corresponding to the (111), (222), and (420) planes of cubic ZnS (JCPDS: 05-0566) [30]. Pristine TiO_2_ displays characteristic peaks at 25.28°, 36.94°, 37.80°, 38.57°, 48.05°, 53.89°, 55.06°, 62.12° and 62.69°, corresponding to the (101), (103), (004), (112), (200), (105), (211), (213) and (204) planes of anatase TiO_2_ (JCPDS: 21-1272) [31], confirming the successful synthesis of pure ZnS and TiO_2_.

The XRD pattern of the TZnS−100 composite (Figure 3a) contains peaks from both anatase TiO_2_ and cubic ZnS, with reflections corresponding to the TiO_2_ (101) plane and ZnS (111), (222), and (420) planes (JCPDS: 21-1272 and 05-0566, respectively). Notably, the ZnS (111) peak slightly shifts to higher angles in the TZnS-X composite compared to pristine ZnS (Figure 3b), indicating lattice strain. This lattice distortion likely results from TiO_2_/ZnS heterostructure formation, potentially involving interfacial charge transfer, and is typical of core–shell structures with intimate interfacial contact [32].

X-ray photoelectron spectroscopy (XPS) was employed to determine the elemental composition and chemical state of the TZnS photocatalyst. The survey spectra (Figure S3a) confirm the presence of Ti, O, and C in TiO_2_; Zn, S, O, and C in ZnS; and Ti, O, Zn, S, and C in the TZnS composite. The C 1s peak at 284.80 eV (Figure S3b) was used for charge calibration, while other signals are attributed to adventitious carbon contamination from instrumental or sample handling.

**Figure 4:**
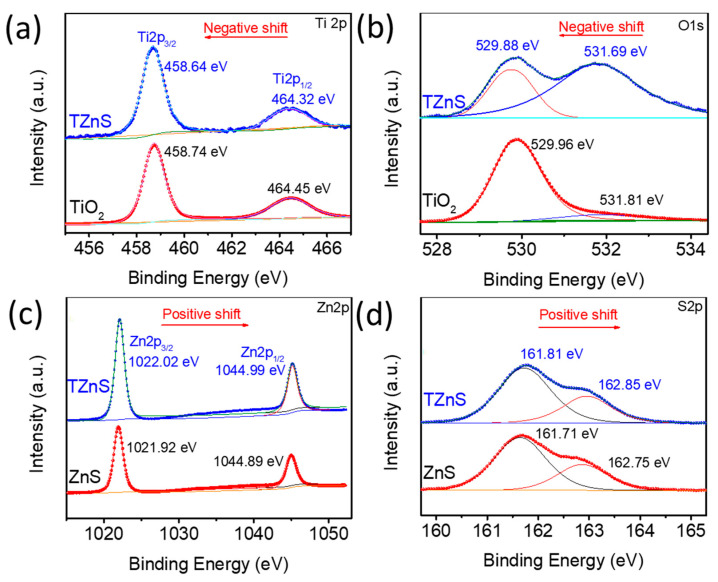
HR-XPS spectra of TZnS−100, TiO_2_ and ZnS: (**a**) Zn 2p; (**b**) S 2p; (**c**) Ti 2p; (**d**) O 1s.

Analysis of the Ti 2p spectrum of TZnS (Figure 4a) shows peaks at 458.64 eV (Ti 2p_3/2_) and 464.32 eV (Ti 2p_1/2_), with a spin–orbit splitting of 5.68 eV, confirming the Ti^4+^ oxidation state, consistent with TiO_2_ XPS references [33]. Compared to pure TiO_2_, the Ti 2p peaks of TZnS shift to lower binding energies, indicating partial Ti^4+^ to Ti^3+^ reduction upon ZnS coupling, generating oxygen vacancies and markedly enhancing photocatalytic oxidation activity [11,14].

In the O 1s spectra (Figure 4b), pure TiO_2_ exhibits two components: a primary peak at 529.96 eV (lattice oxygen, Ti–O) and a secondary peak at 531.81 eV (surface hydroxyl groups, –OH). In TZnS, both Ti–O and –OH peaks shift to lower binding energies compared to pure TiO_2_, suggesting electron transfer from ZnS to TiO_2_ at the heterojunction interface.

Further comparison of the Zn 2p spectrum in the TZnS composite (Figure 4c) shows a slight increase in binding energy compared to pure ZnS, with the Zn 2p_3/2_ peak shifting from 1021.92 eV (pure ZnS) to 1022.02 eV and the Zn 2p_1/2_ peak from 1044.89 eV (pure ZnS) to 1044.99 eV (0.1 eV shift). This positive shift indicates reduced electron density around Zn atoms, consistent with interfacial charge redistribution. Similarly, the S 2p spectrum (Figure 4d) shows a 0.1 eV positive shift relative to pure ZnS [34]. These shifts in Zn and S species indicate electron depletion within ZnS, supporting electron transfer from ZnS to the TiO_2_ at the heterojunction interface, confirming the formation of a TiO_2_/ZnS heterojunction.

### 2.2. Photocatalyst Activity

#### 2.2.1. Visible Light Degradation of TC

**Figure 5:**
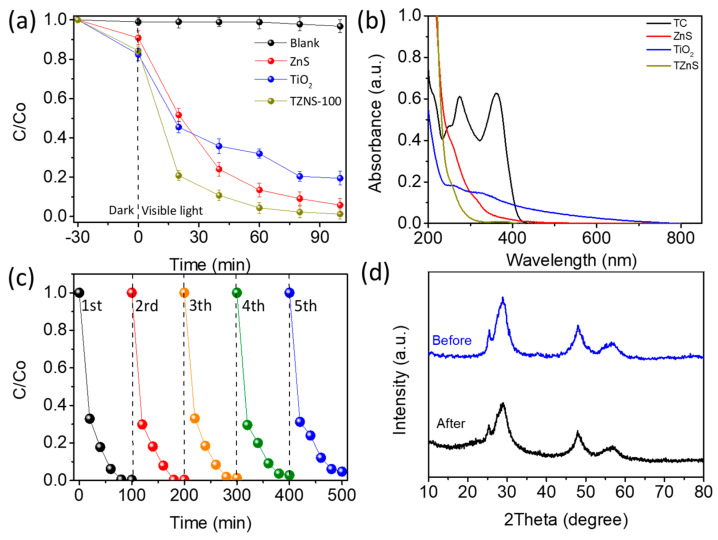
(**a**) Photocatalytic degradation of TC over TiO_2_, ZnS, TZnS−100; (**b**) UV−vis spectra; (**c**) Recyclability test of TZnS−100; (**d**) XRD patterns of TZnS−100 before and after recyclability test. Condition: catalyst concentration: 200 mg/L; TC concentration: 20 mg/L; total volume: 50 mL.

To assess the photocatalytic performance of TiO_2_, ZnS, and TZnS−100, degradation experiments were conducted on a 20 mg/L TC solution under visible light irradiation. Dark adsorption tests confirmed all catalysts reached adsorption equilibrium within 30 min, with no TC self-degradation observed in control experiments (Figure S6). As shown in Figure 5a (−30 min as the dark adsorption equilibrium point and 0 min as the start of irradiation), TZnS−100 achieved 80% TC degradation within 20 min, demonstrating superior activity over pristine TiO_2_ (55% degradation, 1.45 times) and ZnS (50% degradation, 1.60 times). UV-vis spectra after 20 min of visible light irradiation (Figure 5b) show residual TC concentration: TZnS−100 < ZnS < TiO_2_ (at λ = 357 nm, relative to TC control). This enhancement stems from the TiO_2_/ZnS N-N heterojunction, which enhances charge separation kinetics. Interfacial synergy facilitates efficient photogenerated carrier migration through the heterostructure, while maintaining ample surface active sites, as evidenced by the BET surface area trend: TZnS−100 > ZnS > TiO_2_ (Figure S4 and Table S1).

To evaluate recoverability, TZnS−100 was separated after each degradation cycle, washed with deionized water, dried, and reused. After the fifth cycle, TZnS−100 retained over 94% of its initial activity (Figure 5c), for surpassing the 20% drop of pure ZnS (Figure S7a) and 30% drop of pure TiO_2_ (Figure S7b) within fifth cycles, thereby demonstrating outstanding recyclability despite a marginal 6% efficiency decrease, likely due to cumulative catalyst loss during recovery and mechanical wear. Structural stability was verified by XRD (Figure 5d), with unchanged diffraction patterns for TZnS−100 after cycling, indicating excellent phase stability during photocatalytic operation.

#### 2.2.2. Effect of Different Reaction Conditions

**Figure 6:**
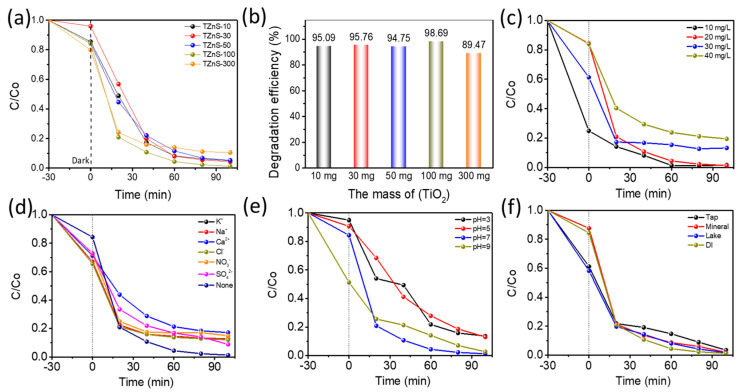
Effects of Different Factors on TZnS−100 Photocatalytic Degradation Efficiency (**a**) As a function of TiO_2_ mass loading; (**b**) TC degradation after 100 min; (**c**) TC concentration; (**d**) Common cation and anion; (**e**) Solution pH; (**f**) Water source (Catalyst concentration: 200 mg/L; TC concentration: 20 mg/L; Reaction volume: 50 mL).

Optimal TiO_2_ Loading for TZnS−100 Synthesis. Systematic optimization identified 100 mg of TiO_2_ as the optimal loading for TZnS synthesis, maximizing TC degradation efficiency (Figure 6a). This results from balanced TiO_2_/ZnS interfacial contact, enhancing heterojunction charge transfer. Excessive loading (300 mg) reduced efficiency by 9% within 100 min (Figure 6b) due to TiO_2_ nanoparticle aggregation, blocking active sites, and causing light scattering.

Concentration Effects. TZnS−100 maintained over 96% TC degradation at ≤20 mg/L (Figure 6c), but efficiency dropped above 30 mg/L. This degradation limitation stems from three interconnected mechanisms: (1) Mass transport limitations reduce TC diffusion; (2) Photon absorption saturation at high pollutant concentrations; (3) Radical scavenging by TC degradation intermediates.

Ionic Interference Resistance. Common ions (${Na}^{+}$, $K^{+}$, ${Cl}^{-}$, and ${SO}_{4}^{2-}$) caused less than 5% efficiency variations (Figure 6d), thereby demonstrating the exceptional ionic interference resistance of the TZnS−100 catalyst.

pH Dependence. Neutral conditions (pH = 7) maximized degradation (99.0%), while acidic (pH = 3) and alkaline (pH = 11) conditions reduced efficiency by 20–40% (Figure 6e) due to surface charge effects: protonation repels TC in acidic conditions, and hydroxide consumes photogenerated holes and ·OH radicals in alkaline conditions.

Water Matrix Performance. TZnS−100 maintained over 96.0% TC removal within 90 min across complex water matrices (Figure 6f), including tap water (96.2%), mineral water (96.5%), and Fuyang Lake water (96.1%). Deionized water showed slightly higher efficiency (99.0%) due to the absence of natural organic matter competing for reactive species and light absorption, confirming resilience to carbonates and silicates.

### 2.3. Possible Photocatalytic Mechanism

#### 2.3.1. Active Species Determination

**Figure 7:**
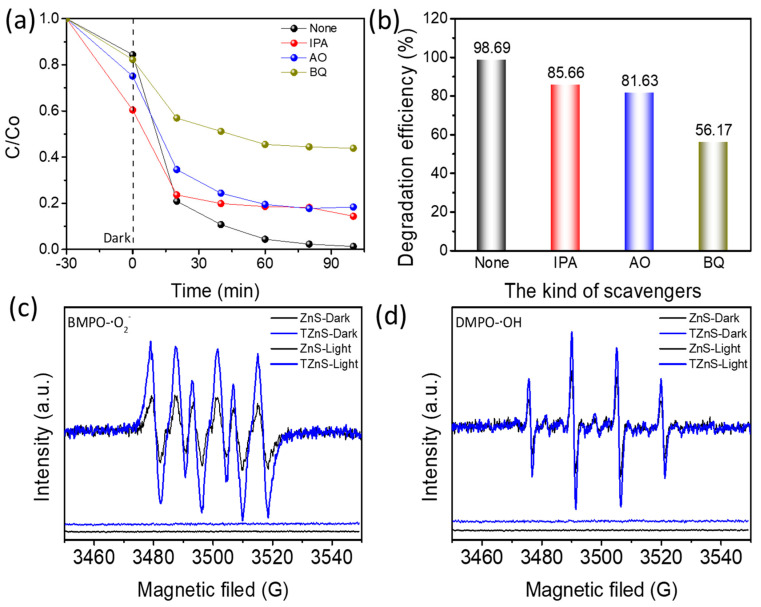
(**a**,**b**) Active species trapping experiments (TZnS−100 concentration: 200 mg/L; TC concentration: 20 mg/L; Total volume: 50 mL), (**c**) DMPO−$\cdot O^{2-}$ and (**d**) DMPO−·OH adduct (1:2:2:1 quartet) ESR spectra under dark and visible−light conditions.

Active species trapping experiments systematically elucidated the photocatalytic degradation mechanism of TZnS−100 for TC (Figure 7a,b). Quenching hydroxyl radicals (·OH) with isopropyl alcohol (IPA) reduced degradation efficiency by 85.7%, indicating that·OH contributes to secondary oxidation via H-atom abstraction. Scavenging holes ($h^{+}$) with ammonium oxalate (AO) caused an 81.6% efficiency loss, demonstrating hole directly oxidizes TC via nucleophilic attack. Quenching superoxide radicals ($\cdot O^{2-}$) with benzoquinone (BQ) resulted in a 56.2% inhibition, confirming $\cdot O^{2-}$ as the primary reactive species due to electrophilic addition to TC’s conjugated systems, amplified by radical chain reactions [20].

ESR spectroscopy with DMPO spin-trapping agents confirmed the photocatalytic generation of reactive oxygen species in ZnS and TZnS−100 heterostructure [35]. Under visible-light irradiation from a xenon lamp, distinct DMPO-$\cdot O^{2-}$ and DMPO-·OH signals appeared in the 3455–3555 mT magnetic field range (Figure 7c,d), absent in dark conditions, establishing the photo-driven radical formation.

**Figure 8:**
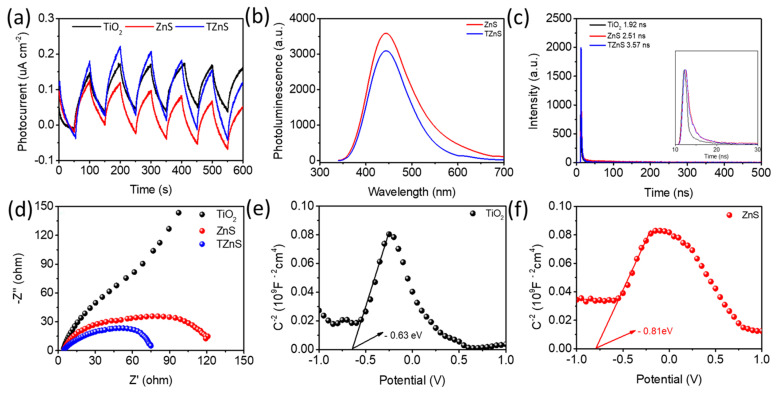
(**a**) Photocurrent response; (**b**) Photoluminescence spectra; (**c**) Time−Resolved Photoluminescence; (**d**) Electrochemical Impedance Spectroscopy; (**e**,**f**) Mott−Schottky plots of TiO_2_, ZnS, and TZnS−100.

Importantly, the DMPO-$\cdot O^{2-}$ signal intensity in TZnS−100 significantly exceeded that observed for pristine ZnS, indicating enhanced superoxide radical ($\cdot O^{2-}$) production resulting from interfacial charge separation [36]. Specifically, photogenerated holes ($h^{+}$) preferentially migrate toward ZnS due to its higher valence band position (−0.81 eV vs. NHE vs. −0.63 eV for TiO_2_), thereby reducing electron-hole recombination in the TiO_2_ core. This prolonged electron lifetime (>3.2 ns, measured by time-resolved photoluminescence, Figure 8c) facilitates efficient oxygen reduction reactions (O_2_ +$\;e^{-}$ → $\cdot O^{2-}$) at the TiO_2_ interface.

Additionally, a weaker ESR signal of DMPO-·OH over ZnS was observed compared with TZnS−100, suggesting a limited generation of hydroxyl radicals in the photocatalytic reaction. These results confirm the synergistic effect of carrier separation in the TiO_2_/ZnS heterojunction, promoting TC photocatalytic degradation, which is consistent with active species trapping results.

#### 2.3.2. Charge Transfer Kinetics

To systematically elucidate interfacial charge dynamics in the TZnS−100 heterostructure, complementary optoelectronic characterizations were hierarchically conducted under visible-light irradiation, establishing a progressive evidence chain for enhanced interfacial charge transfer.

First, transient photocurrent analysis (Figure 8a) confirmed the overall charge separation capability of the heterostructure. Notably, TZnS−100 exhibited the highest transient photocurrent density, surpassing that of pure ZnS and pure TiO_2,_ respectively, demonstrating superior charge generation and interfacial transfer efficiency.

Subsequently, steady-state photoluminescence (PL) spectroscopy, performed using a FluoroMax-4 fluorescence spectrophotometer equipped with a 500 W xenon lamp light source (Figure 8b; λ = 320 nm), provided direct evidence of recombination suppression. Specifically, the lower PL intensity in TZnS−100 corroborates reduced electron-hole recombination through interfacial charge separation, thereby reinforcing the photocurrent findings.

Furthermore, time-resolved PL (TRPL, Figure 8c) quantitatively validated recombination kinetics. TZnS−100 ($\tau_{avg}\approx 3.57\;ns$) exhibited an average carrier lifetime 42% longer than ZnS ($\tau_{avg}\approx 2.51\;ns$) and 86% longer than TiO_2_ (1.92 ns), indicating significantly prolonged carrier availability. Critically, these data not only align with PL quenching trends but also establish that the TiO_2_/ZnS heterojunction suppresses recombination.

Consequently, electrochemical impedance spectroscopy (EIS, Figure 8d) mechanistically explained the observed phenomena. The smaller Nyquist arc radius in TZnS−100 unequivocally indicates minimized charge-transfer resistance, originating from interfacial synergy in the TiO_2_/ZnS N-N heterojunction [37], which simultaneously: (1) facilitates rapid carrier transport; (2) reduces radiative recombination; and (3) ultimately yields amplified photocatalytic efficiency.

To elucidate the photocatalytic mechanism, the band structure of TiO_2_, ZnS, and TZnS−100 was systematically characterized. Tauc plot analysis of UV–vis absorption spectra (Figure S5a) revealed distinct band gap variations: pristine TiO_2_ exhibited a band gap of 3.15 eV, and pure ZnS showed 3.30 eV [38]. Correspondingly, Figure S5b demonstrates a 12 nm blue shift in the absorption edge of TZnS−100 relative to TiO_2_, primarily attributed to the quantum confinement effect of the deposited ZnS nanoparticles [39]. This shift reflects successful TiO_2_/ZnS heterojunction integration, elevating the conduction band minimum energy, consistent with density functional theory (DFT) calculations confirming increased effective masses at the heterojunction boundary [40].

**Figure 9:**
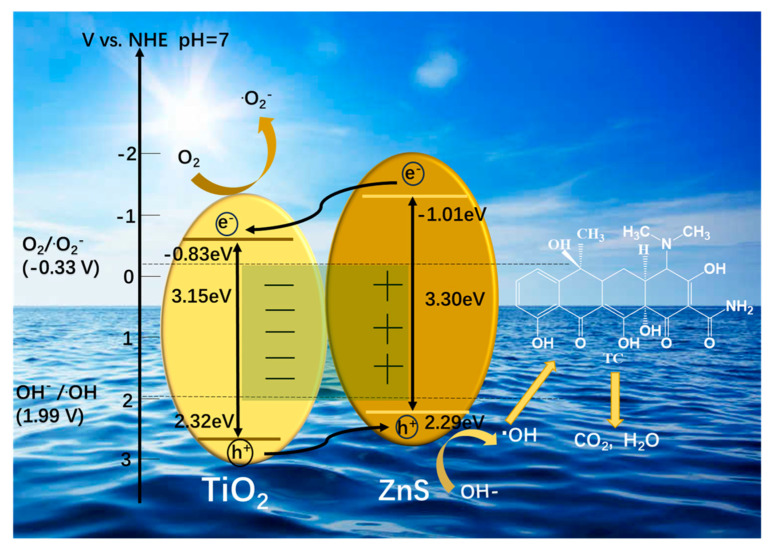
Schematic diagram of the photocatalytic degradation mechanism of TC by TZnS−100.

Mott–Schottky analysis quantitatively determined the interfacial energetics of the semiconductor components. The characteristic positive slopes in Mott–Schottky plots (Figure 8e,f) conclusively verify n-type behavior for both anatase TiO_2_ and synthesized ZnS [41,42]. The flat band potentials of anatase TiO_2_ and ZnS are −0.63 V and −0.81 V (vs. NHE, pH = 7; vs. Ag/AgCl), respectively. For n-type semiconductors, the $E_{fb}$ resides approximately 0.2 V negative relative to the conduction band minimum ($E_{CB}$) due to pH-dependent Fermi level pinning. Using the relationship $E_{VB}={\;E}_{g}\;+{\;E}_{CB}$ [43], the conduction band (CB) and valence band (VB) energies for anatase TiO_2_ were calculated as −0.83 eV/2.32 eV, and for ZnS as −1.01 eV/2.29 eV. The resulting band alignment diagram (Figure 9) reveals a type II heterojunction between TiO_2_ and ZnS, facilitating efficient separation of photogenerated charge carriers through their staggered band offsets [44].

#### 2.3.3. Photocatalytic Mechanism

The photocatalytic mechanism of the TZnS−100 heterojunction, consistent with experimental characterizations, is illustrated in Figure 9. The N-N type heterojunction formed between TiO_2_ and ZnS exhibits optimal band alignment. Under visible light irradiation, photogenerated electron—hole pairs are produced in both semiconductors through excitation from the valence band (VB) to the conduction band (CB), facilitating effective charge separation via the type—II heterojunction structure [45,46]. The favorable band alignment at the TiO_2_/ZnS heterojunction promotes efficient interfacial charge separation: photogenerated electrons transfer from the ZnS CB to the TiO_2_ CB, while holes remain in the ZnS VB. This spatial separation induces an interfacial electric field, significantly suppressing electron-hole recombination, as evidenced by the enhanced photocurrent response (Figure 8a) and reduced charge transfer resistance in EIS (Figure 8d). Within the reaction system, electrons accumulated in the TiO_2_ CB participate in oxygen reduction to form superoxide radical anions ($\cdot O^{2-}$): TiO_2_ (_CB_) $e^{-}+O_{2}\;\to \;\cdot O_{2}^{-}$. Meanwhile, holes in the ZnS VB drive water oxidation to generate hydroxyl radicals (·OH): ZnS (_VB_) $h^{+}+H_{2}O\to \cdot OH+{OH}^{-}$. The synergistic action of $\cdot O^{2-}$ and ·OH induces stepwise degradation of TC: the radical initially attacks the aromatic ring, disrupting the conjugated π systems, followed by ring-opening reactions to form intermediates, ultimately mineralizing into nontoxic inorganic products such as CO_2_ and H_2_O. Therefore, the constructed TiO_2_/ZnS heterojunction conforms to the conventional type—II charge transfer mode.

## 3. Experimental Section

### 3.1. Chemicals

Anhydrous ethanol (C_2_H_5_OH), tetrabutyl titanate (TBT), zinc acetate dihydrate (Zn(CH_3_COO)_2_·2H_2_O), thiourea (CH_4_N_2_S), hexadecyltrimethylammonium bromide (CTAB, 99%), nitorblue tetrazolium chloride (NBT), t-BuOH, tetracycline hydrochloride (TC), and triethanolamine (TEOA) were purchased from Sinopharm Chemical Reagent Co., Ltd. (Shanghai, China). All chemicals were used as received without further purification.

### 3.2. Synthesis of TiO_2_/ZnS

The hollow TiO_2_ sphere was synthesized via template-directed self-assembly, following our previously established methods [47,48]. Initially, 3 g of tetrabutyl titanate and 2 g of cationic polystyrene spheres (CPS) were added to 50 mL of ethanol and vigorously stirred for 24 h. Subsequently, a solution containing 1 mL of H_2_O and 9 mL of ethanol was introduced to the reaction system to initiate the hydrolysis reaction, which proceeded at an ice bath for 24 h. Finally, the samples were washed with deionized water and ethanol, dried at 60 °C for 3 h, and then calcined at 450 °C for 2 h to yield the hollow TiO_2_ spheres.

The TiO_2_/ZnS composite was synthesized using a hydrothermal reaction with pre-prepared TiO_2_ as the substrate. First, TiO_2_ (10, 30, 50, 100, or 300 mg) and 0.01 mol of Zn(CH_3_COO)_2_·2H_2_O were dispersed in 10 mL of ethanol using sonication for 30 min to prepare solution A. Secondly, 0.01 mol of CTAB and 0.01 mol of thiourea (CH_4_N_2_S) were added to 40 mL of ethanol and sonicated for 10 min to form solution B. Solution A was slowly introduced into solution B using an injection method (10 μL per second). The resulting mixture was vigorously stirred for 120 min, before being transferred to a 100 mL sealed autoclave and heated at 140 °C for 4 h.

Finally, the samples were washed three times with deionized water and ethanol, and then dried at 60 °C to remove residual solvent, yielding the TiO_2_/ZnS composite samples (denoted as TZnS-X, where X corresponds to the initial TiO_2_ mass: 10, 30, 50, 100, 300).

Pure ZnS catalysts were prepared using the same method without adding TiO_2_.

### 3.3. Photocatalyst Characterization

The microscopic morphology and structure of the TZnS photocatalysts were characterized using scanning electron microscopy (SEM, JEOL JSM-7001F, Tokyo, Japan) and transmission electron microscopy (TEM, JEOL JEM-210HR). The crystal composition and crystallization degree of the catalysts were determined by X-ray diffraction (XRD, Rigaku D/max2500V, Tokyo, Japan). The light absorption range of the TZnS photocatalyst was determined using a UV-visible diffuse reflectance spectrometer (DRS, Shimadzu UV-2600, Kyoto, Japan) with a scanning range of 200—800 nm. The surface elemental state of the photocatalyst was analyzed using X-ray photoelectron spectroscopy (XPS, AMICUS, Berkshire, UK). Finally, the photogenerated radical signals were assessed using an electron paramagnetic resonance instrument (EPR, Bruker ESR 300, Billerica, MA, USA).

### 3.4. Photocatalytic Activity Test

The photodegradation experiment utilized the TZnS photocatalyst to treat a tetracycline hydrochloride (TC) solution (20 mg/L) as the target antibiotic contaminant. Visible light irradiation (λ > 420 nm) was provided by a 300 W xenon lamp (Beijing Perfectlight PLS-SXE300, Beijing, China). Experimental procedure: Initially, 10 mg of TZnS catalyst was added to 50 mL of the TC solution and sonicated for 3 min. The suspension was then magnetically stirred in the dark for 30 min to establish adsorption—desorption equilibrium. Subsequently, the xenon lamp was activated to initiate visible—light—driven photodegradation. At predetermined intervals, 2 mL aliquots were sampled and immediately filtered through a 0.22 μm filter to obtain a clear solution. The TC degradation efficiency was measured by analyzing the residual solution’s absorbance at 357 nm using a UV-Vis spectrophotometer (Shimadzu UV-2600). After each experiment, the remaining catalyst was filtered, washed, and dried for subsequent cycle tests.

### 3.5. Electrochemical Testing

Electrochemical measurements were conducted in a 0.5 M Na_2_SO_4_ electrolyte using a CHI660B electrochemical workstation (CH Instruments, Austin, TX, USA) with a standard three-electrode system. A platinum sheet served as the counter electrode, an Ag/AgCl reference electrode (saturated KCl) was employed for potential measurement, and ITO-coated glass (1 cm^2^ active area) served as the working electrode.

The working electrode was prepared as follows: 5 mg of TZnS active material was uniformly dispersed in a mixed solvent of 0.1 mL 5 wt% Nafion solution and 0.5 mL ethylene glycol, followed by sonication for 30 min to ensure homogeneity. A 40 μL aliquot of the suspension was precisely dispensed onto the ITO substrate using a micropipette, followed by thermal annealing at 60 °C for 8 h to form a uniform thin film. The electrode was cooled to room temperature before electrochemical testing.

## 4. Conclusions

In summary, we developed a rationally designed TZnS−100 heterojunction photocatalyst optimized for enhanced TC degradation under visible light. The TZnS−100 composite demonstrated superior photocatalytic activity, achieving 80% TC degradation within 20 min, representing 1.55−fold and 1.50−fold efficiency improvements compared to pristine TiO_2_ and ZnS, respectively. In addition, the optimal photocatalytic performance was achieved by incorporating 100 mg of TiO_2_ (prepared via the hydrothermal method) into the TZnS−100 composite. Time-resolved photoluminescence spectroscopy confirmed that the TiO_2_/ZnS heterojunction significantly prolonged charge carrier lifetimes (TZnS−100$\;\tau_{avg}\approx 3.57\;ns$ vs. TiO_2_:$\;\tau_{avg}\approx 1.92\;ns$), indicating efficient separation of photo-generated carriers. Further evidence from ESR experiments revealed enhanced generation of superoxide radicals ($\cdot O^{2-}$) under visible light irradiation (DMPO−$\cdot O^{2-}$ quartet signal), with negligible radical activity in dark conditions. Moreover, the photocatalytic mechanism was determined to proceed via a type−II heterojunction model. This work not only provides a promising strategy for designing hollow heterojunction photocatalysts but also offers in−depth mechanistic insights into the critical role of superoxide radical anions in the photocatalytic degradation, providing theoretical guidance for the design of efficient photocatalytic systems.

## Data Availability

No new data were created or analyzed in this study. Data sharing is not applicable to this article.

## Supplementary Materials

The following supporting information can be downloaded at: https://www.mdpi.com/article/10.3390/molecules30173644/s1, Figure S1: (a–f) TEM, the corresponding element mappings, HRTEM images of TiO_2._ Figure S2: (a–f) TEM, the corresponding element mappings, HRTEM images of ZnS. Figure S3: HRXPS spectra of T, Z and TZ: (a) full spectra, (b) C1s. Figure S4: BET spectra of (a) TiO_2_, ZnS, TZnS, and (b) TZnS-X (X = 10, 30, 50, 100, 300). Table S1: Surface area TiO_2_, ZnS and TZnS-X (X = 10, 30, 50, 100, 300). Figure S5: (a) DRS spectra and (b) Band gad of TiO_2_, ZnS, TZnS. Figure S6: Adsorption studies for TiO_2_, ZnS and TZnS under dark irradiation: Catalytic conditions with a catalyst concentration of 200 mg/L and a target compound (TC) concentration of 20 mg/L. Figure S7: Recyclability test of (a) ZnS and (b) TiO_2._
