# Supplementary material for: Construction of Hollow TiO2/ZnS Heterojunction Photocatalysts for Highly Enhanced Photodegradation of Tetracycline Hydrochloride
Source: Molecules. 2025 Sep 7;30(17):3644. doi: 10.3390/molecules30173644 (PMC12430114; doi:10.3390/molecules30173644)
Supplement: Supplementary file 1 [file molecules-30-03644-s001.zip › molecules-3826949-supplementary.pdf]

Construction of Hollow TiO_2/ZnS Heterojunction Photocatalysts for Highly Enhanced Photodegradation of Tetracycline Hydrochloride

Ying Zhang *, Anhui Su, Yuqin Ding, Yuhan Wu, Yapeng Tan and Jianguo Chang *

Anhui Provincial Key Laboratory of Green Carbon Chemistry, School of Chemistry and Material Engineering, Fuyang Normal University, Fuyang 236037, China

* Correspondence: zhangying@fynu.edu.cn (Y.Z.); jgchang@mail.ustc.edu.cn (J.C.)

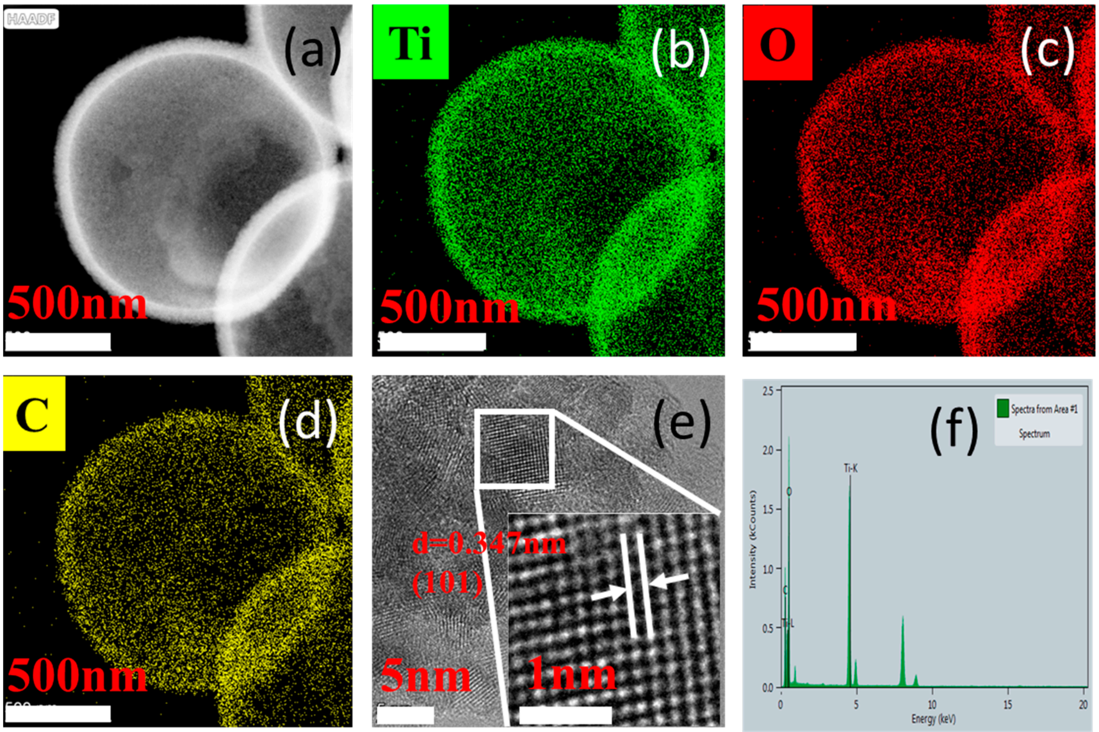

Figure S1. (**a**–**f**) TEM, the corresponding element mappings, HRTEM images of TiO_2

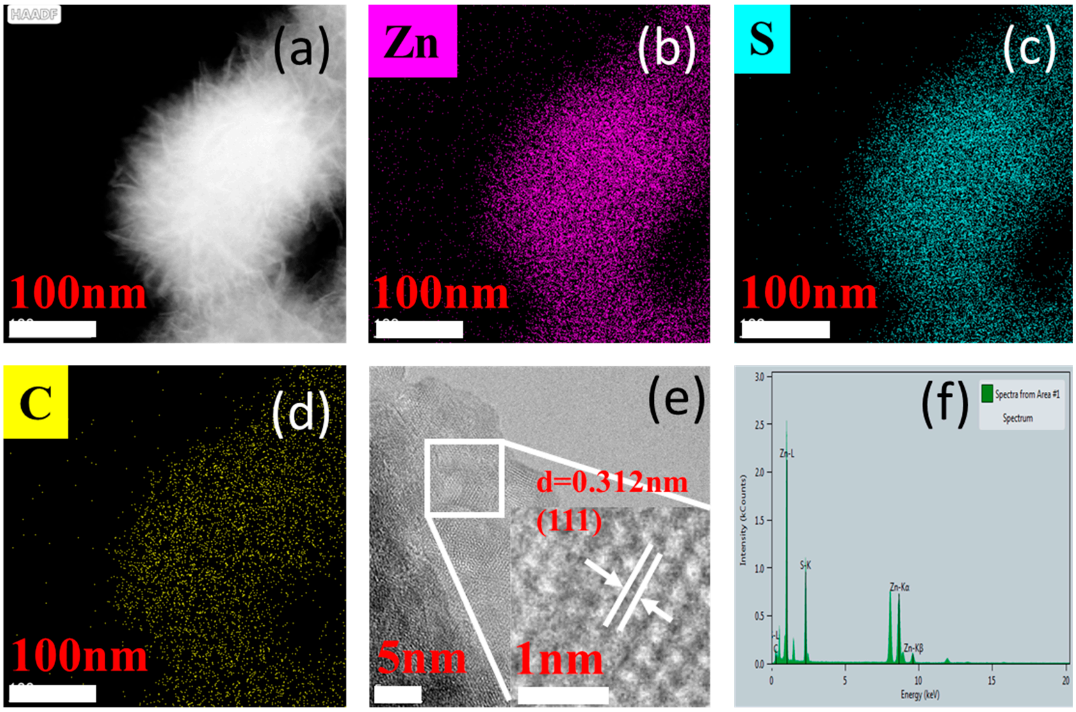

Figure S2. (**a**–**f**) TEM, the corresponding element mappings, HRTEM images of ZnS

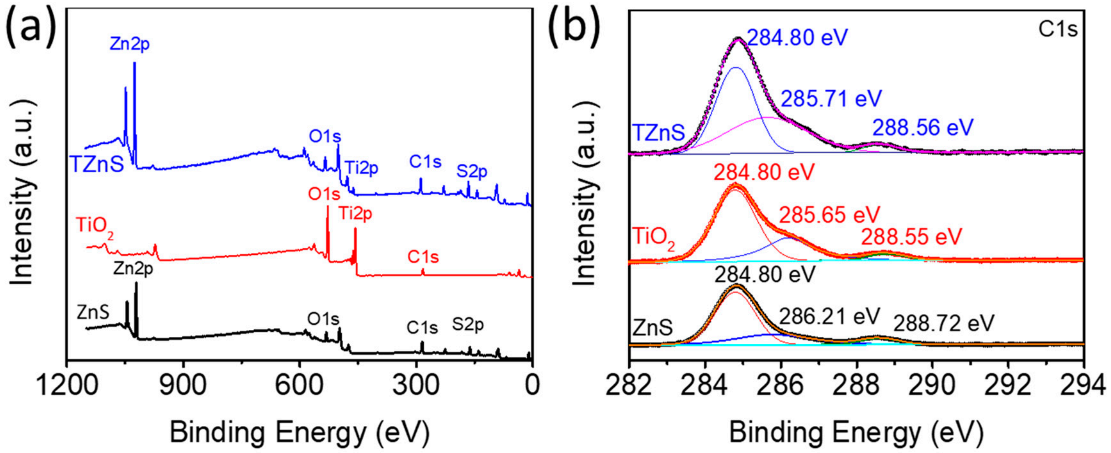

Figure S3. HRXPS spectra of T, Z and TZ: (**a**) full spectra, (**b**)C1s

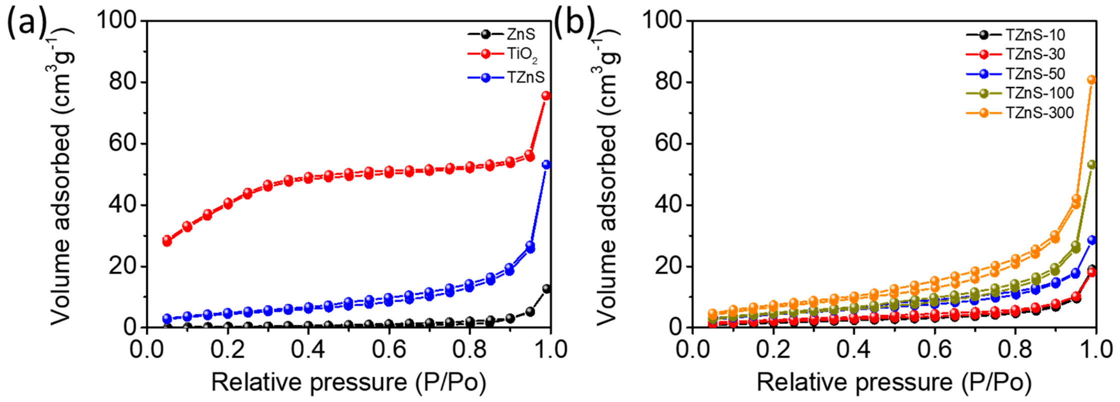

Figure S4. BET spectra of (**a**) TiO_2, ZnS, TZnS, and (**b**) TZnS-X (X=10,30,50,100,300)

Table S1. Surface area TiO_2, ZnS and TZnS-X (X=10,30,50,100,300)

Sample	BET (m^2/g)
TiO_2	12.619
ZnS	3.311
TZnS-10	5.683
TZnS-30	5.750
TZnS-50	12.811
TZnS-100	17.338
TZnS-300	27.278

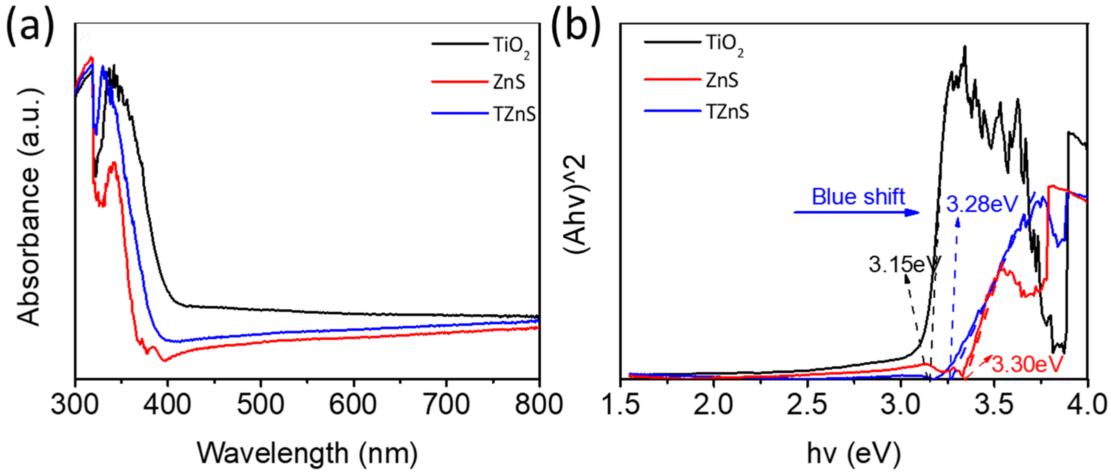

Figure S5. (a) DRS spectra and (b) Band gad of TiO_2, ZnS, TZnS

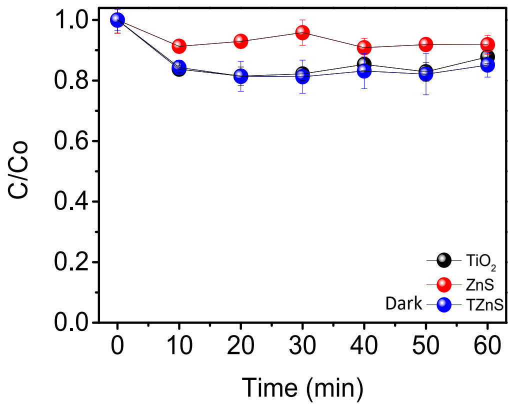

Figure S6. Adsorption studies for TiO_2, ZnS and TZnS under dark irradiation: Catalytic conditions with a catalyst concentration of 200 mg/L and a target compound (TC) concentration of 20 mg/L

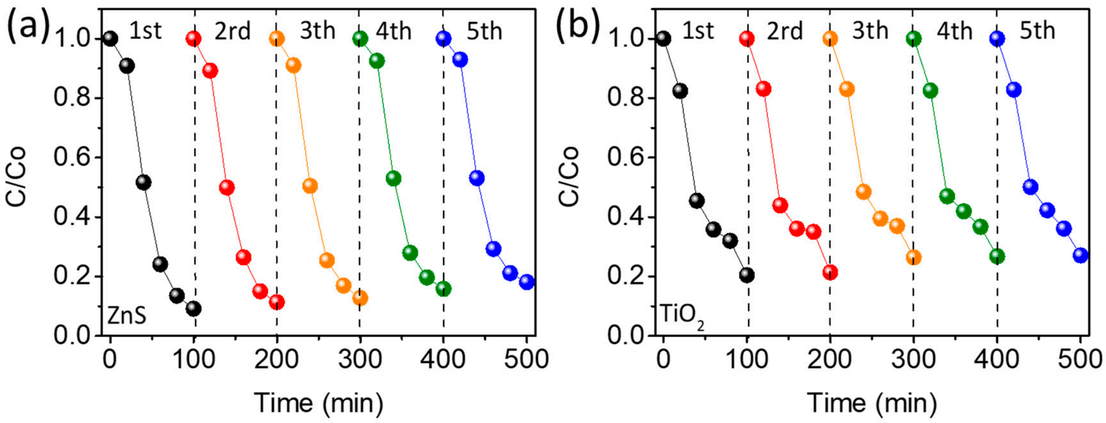

Figure S7. Recyclability test of (**a**) ZnS and (**b**) TiO_2
